# Experimental and Computational Approaches to Direct Cell Reprogramming: Recent Advancement and Future Challenges

**DOI:** 10.3390/cells8101189

**Published:** 2019-10-02

**Authors:** Rihab Gam, Minkyung Sung, Arun Prasad Pandurangan

**Affiliations:** MRC Laboratory of Molecular Biology, Francis Crick Avenue, Cambridge CB2 0QH, UK; msung@mrc-lmb.cam.ac.uk (M.S.); apandura@mrc-lmb.cam.ac.uk (A.P.P.)

**Keywords:** transdifferentiation, direct reprogramming, computational biology, regenerative medicine, cell therapy

## Abstract

The process of direct cell reprogramming, also named transdifferentiation, permits for the conversion of one mature cell type directly into another, without returning to a dedifferentiated state. This makes direct reprogramming a promising approach for the development of several cellular and tissue engineering therapies. To achieve the change in the cell identity, direct reprogramming requires an arsenal of tools that combine experimental and computational techniques. In the recent years, several methods of transdifferentiation have been developed. In this review, we will introduce the concept of direct cell reprogramming and its background, and cover the recent developments in the experimental and computational prediction techniques with their applications. We also discuss the challenges of translating this technology to clinical setting, accompanied with potential solutions.

## 1. Introduction

**Figure 1:**
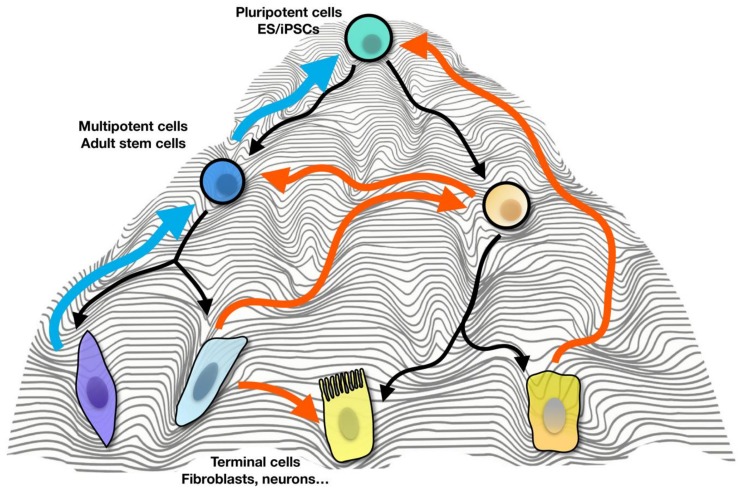
Cell fate plasticity and the epigenetic landscape currently applied for direct cell reprogramming. Pluripotent cells, including embryonic stem cells (ES) and induced pluripotent stem cells (iPSCs) can differentiate into any type of multipotent or adult cells (black arrows) which in turn can differentiate into terminal cells (e.g., fibroblasts, neurons, and astrocytes). This can happen naturally during their development or in response to external factors if done in vitro. The paths which takes either a differentiated cell or a multipotent cell back to the pluripotent/stem cell state is shown here in blue arrows. Transdifferentiation (orange arrows) is the process by which the terminally differentiated cell or adult cell can be converted into any other terminally differentiated cell or adult cell without passing by a pluripotency state. Differentiated cells can also be directly converted into the pluripotency state via the process of transdifferentiation.

The epigenetic landscape model proposed by Conrad Waddington in 1957 provided a framework to explain cellular differentiation through epigenetic changes rather than genetic inheritance [1]. In this model, a pluripotent cell takes a complex path defined by ridges and valleys on the developmental landscape to reach a final fully-differentiated and specialized cell (Figure 1). For the first time the possibility of cloning a vertebrate was shown through the nuclear transfer experiments in *Xenopus laevis* that the differentiated cells preserve their complete genetic material and it led to the understanding that the state of a fully differentiated cell could be reverted back to a dedifferentiated state [2]. For the first time, this opened up the field of cell rejuvenation and reprogramming. Further studies followed on to add more body of evidence supporting the concept of cell reprogramming [3,4,5]. However, the mechanism behind the cell state conversion was unclear until it was shown by Takahashi and Yamanaka that a set of key transcription factors are required to convert a differentiated cell to an induced pluripotent stem cell (iPSCs) [6].

Traditionally, the conversion of somatic cells from one specific type to another relied on a successful conversion to an iPSCs which uses the mechanism of epigenetic regulations to remodel somatic cells by resetting its chromatic structure and the methylation states of histone proteins and DNA [7]. In the past, various experimental techniques been developed to generate iPSCs. They include somatic cell transfer into oocytes, cell fusion of somatic cells and iPSCs and the reprogramming of somatic cells by inserting cell extracts from pluripotent stem cells [8,9,10,11,12]. Various types of cells including fibroblast, keratinocytes, melanocytes, hepatocytes, astrocytes, neural stem cells, T cells, blood stem cells, and urine cells have been reprogrammed to iPSCs [13].

The iPSCs possess similar properties of an embryonic stem cells (ES) which can differentiate into any other cell type. This allowed the possibility of using iPSCs as well as ES for various clinical applications including cell-based therapy, tissue repair, degenerative diseases, aging and cancer [14,15,16,17,18,19].

Despite their potential for clinical applications, the use of iPSCs have raised various concerns including the cost, low efficiency and the duration of conversion due to complex conversion protocols. In addition, the use of iPSC technology in human cell therapy is tightly regulated due to the risk of genetic abnormalities, tumorigenicity, and immunogenicity in the transplanted cells [20]. These drawbacks have limited the usage of iPSCs widely. 

In order to address the issues related to iPSCs, direct cell reprogramming methods were developed. They avoid the need for a pluripotent state while converting a functional cell type from one lineage to another lineage [21]. One of the early examples of direct reprogramming technique identified the overexpression of a key transcription factor called Myod that was responsible for the conversion of fibroblast into myoblast [3]. Since then, the field of direct reprogramming progressed rapidly with a substantial increase in the number of different cell types being covered in human and mice [22]. Unlike iPSCs, direct reprogramming methods does not require cell division which reduces the risk of tumorigenesis. The conversions using direct reprogramming are relatively faster because of bypassing pluripotent cell state and offers great potential for clinical and therapeutic applications [23]. Most direct reprogramming methods either use exogenous transgene overexpression, endogenous gene regulation or pharmacological agents to regulate key reprogramming factors involved for the transdifferentiation process. 

Recent advances in the sequencing technologies and the availability of wealth of data on gene expression profiles of various cell types and good quality biological networks have led to the development of computational prediction methods that complement the experimental technique of direct cell reprogramming. Few computational methods have been developed to predict the key transcription factors required to achieve direct cell conversion [24,25,26,27]. The individual methods have been compared and reviewed elsewhere [28].

**Figure 2:**
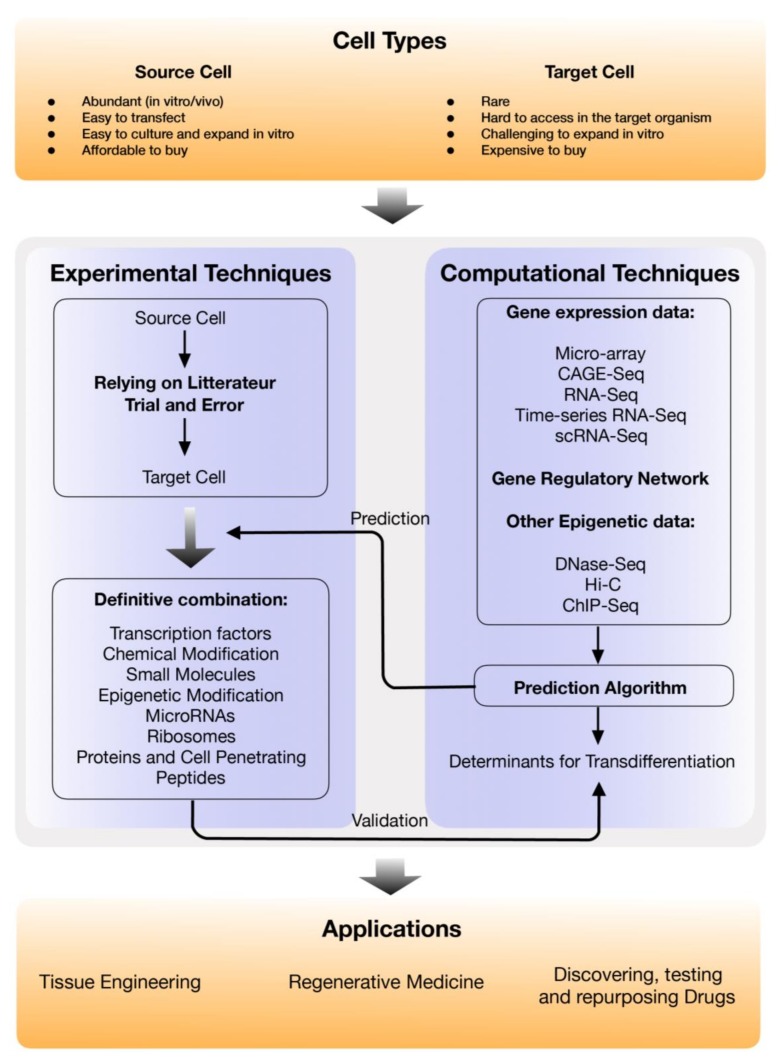
Current and future developments in direct cell reprogramming using experimental and computational techniques and its potential applications. The motivation for cell reprogramming starts with a need in the field for specific types of start and target cells each defined by a set of characteristics stated above in the “Cell Types” Box. Once the choice is made, then a bridge between the experimental and the computational benches is needed (instead of the old method of trial and error) allowing for both to discover and later on validate a definitive list of components needed to reach the target cell to answer the scientific question at hand. The latter is described in the “Experimental techniques” and the “Computational Techniques” boxes. A gamut of applications become possible once this functional framework is established (“Applications” box).

Here, we review various experimental and computational techniques used to achieve direct cell reprogramming and we elaborate on their applications in tissue engineering and regenerative medicine. We also discuss the current challenges and solution to move this technology to clinics. Overview of the techniques, advancement and applications of direct cell reprogramming covered in this review are illustrated in Figure 2.

## 2. Experimental Methods for Direct Cell Reprogramming and the Applications

In vitro transdifferentiation in mice and humans can be guided both by expressing specific transcription factor(s) which are known to play key roles in the maintenance and regulation of the cell state and functions [22]. In order to achieve the cell identity shift, various methods have been developed based on stable and transient expression systems. 

Cell transfection is a method that allows for the expression of either exogenic DNA or RNA in a host cell and can be either transient or stable. 

Transient transfection allows the exogenic DNA or RNA to be expressed without needing to be integrated into the host genome. This can be achieved using various non-viral techniques including liposome and non-liposome mediated transfection and dendrimer-based transfection and electroporation. In liposome-mediated transfection, which as the name states, depends on the transient DNA or RNA to be encapsulated by the liposome for entry into the cell’s cytoplasm [29]. In non-liposomal transfection use lipids and polymers instead of the liposomal encapsulation, these techniques are based on making droplet-like complexes between the foreign nucleic acid and these lipids which can fuse with the cytoplasmic membrane of the host cells allowing the release of the DNA or RNA into the cytoplasm of the cell [30]. In dendrimer-based transfection and electroporation, temporary pores are opened in the cytoplasmic membrane of the cell for the exogenic material to enter the cytoplasm [31].

Unlike transient transfection, stable transfection allows for the exogenic material to be inserted into the host genome and thus expressed permanently. To achieve the latter, techniques like microinjection, which works by injecting foreign DNA or RNA in a cell of choice [32], and virus-mediated gene delivery, which relies on the native machinery of the virus to integrate into the host genome and subsequently transduce the cell [33]. Naturally, the viral genome has all the needed components for it to replicate itself once it infects the host cell. However, with the growing needs of the field, and the safety concerns, researchers adapted viruses using plasmid-based techniques, allowing to introduce plasmids into a host cell to generate viruses instead of a virus infecting a cell to produce more viruses. This has made it possible for the viral genome to be edited via molecular cloning and thus give rise to modified versions capable of directing a wide array of genetic functions within the host cell [34].

Viral vectors can be of two types: Integrating and non-integrating. Upon recognition by the specific receptors on the cell surface, the content of the viral capsid is injected into the cytoplasm of the host cell, from there the viral genetic material can either be translocated into the nucleus to be integrated in the host genome permanently in the case of integrating viruses, or remain in the cytoplasm in the case of non-integrating viral systems [35]. The choice of the appropriate viral vector depends on the purpose of the research, e.g., Lentiviruses are capable of infecting dividing and non-dividing cells by integrating into the host genome and thus, allowing for a stable expression of the genetic material they hold; γ-Retrovirus also allow for a stable expression via integrating into the host genome but are only capable of infecting dividing cells only; Adenoviruses on the other hand, are non-integrating viral systems, capable of infecting dividing and non-dividing cells with a transient expression; like adenoviruses, adeno-associated viruses (AVV) can also infect dividing and non-dividing cells and have a transient expression and are known to not integrate into the host cell genome except a very low frequency of recombinant AVVs integrating the genome of the target cells [36,37].

All of the above viral systems are designed to take up the edited viral genome alongside the needed inserts and markers permitting for the monitoring, quantification, and validation of the change of cell fate during a transdifferentiation process. Perhaps the most interesting inserts currently used in the field of direct reprogramming are the genes coding for transcription factors, these will be discussed in the next section of this review.

### 2.1. Transcription Factors

Transcription factors have been shown to be the tool of choice to be overexpressed in the starting cell type to achieve the change of cell fate. 

In 2012, Heinrich and colleagues succeeded in converting astrocytes into GABAergic or glutamatergic neurons [38], while Karow and colleagues showed that pericyte-derived cells of the adult human brain can be converted to neurons [39]. Endothelial cells were successfully converted from amniotic cells, and monocytes from neural stem cells [40,41]. In 2013, Hendry and colleagues used TFs to directly convert proximal tubule cell line HK-2 into a nephron progenitor [42]. In 2015, Lamper and colleagues performed an elegant study demonstrating that pancreatic exocrine cells can transdifferentiated into pancreatic β-like cells [43]. 

Fibroblast are probably the most popular source cell for the process of transdifferentiation. Kajimura and colleagues converted fibroblasts into functional adipocytes [44], while both Nam et al., and Wada et al., converted them into cardiomyocytes [45,46]. Hiramatsu et al., also used fibroblasts as the source cell and efficiently converted them into chondrocytes [47]. Fibroblasts were also shown to be capable of transdifferentiating into endothelial cells [48], hemogenic endothelial-like precursor cells [49], hematopoietic progenitor cells [50,51], macrophages [52], neurons [53,54,55,56] or hepatocytes [57,58,59] using TFs.

Unlike in-vitro transdifferentiation, the in vivo conditions favor the natural environment of the cells and eliminates the complex process of cell culture and cell transplantation. In vivo, it was demonstrated that the expression of Gata4, Mef2c, and Tbx5 can convert mouse cardiac fibroblasts into functional cardiomyocyte-like cells [60], whereas the expression of the same TFs in vitro failed to do the same [61,62]. 

Pancreatic exocrine cells were also converted into β-cells in vivo [62], Sox9+ cells in liver were successfully transdifferentiated into insulin-secreting ducts [63], non-myocytes into cardiomyocyte-like cells [45], astrocytes into neurons [64] or neuroblasts [65], embryonic and early postnatal callosal projection neurons located in layer II/III were directly reprogrammed into corticofugal projection neurons in layer V/VI [66], and lastly, B cells were converted into hematopoietic stem or progenitor cells [67].

### 2.2. Chemical Modification and Small Molecules

Techniques based on chemical modification and the use of small molecules have revolutionized the cell replacement therapies and drug screening. Compared to other methods, small molecules bring the advantage of structural versatility, relatively cheap to upscale the production and fairly easy to control dosage and timing.

Histone deacetylase inhibitors and DNA methyltransferase inhibitors were the first to be used to facilitate the generation of iPSCs [68]. Later, several other compounds which were shown to successfully mediate mature cell reprogramming as well as replacing the use of other factors [69,70,71].

In 2010, Oct4 in combination with a selection of chemical compounds were used to transdifferentiate somatic cells into iPSCs [72,73] this could not be achieved easily when using just chemical compounds. 

The use of small molecules during the process of transdifferentiation reduces the risk of genetic manipulation when used under clinical setting. Small molecules have been shown to regulate the target gene expression profiles using various mechanisms elegantly reviewed by Yu and colleagues [74].

In 2015, the successful transdifferentiation of fibroblasts into murine and human neurons using only chemical compounds have been reported [75,76]. Li et al., used the Ascl1 and 4 small molecules (Forskolin, ISX9, CHIR99021, and SB431542) to induce neuronal fates from mouse fibroblasts [76] which was enhanced by adding I-BET151 [76]. Hu et al., discovered that VPA, CHIR99021, and Repsox (VCR) can induce neuronal progenitor cells from mouse and human somatic cells [75,76]. In the nervous system, a cocktail of 9 chemical compounds (LDN193189, SB431542, TTNPB, Thiazovivin, CHIR99021, VPA, DAPT, Smoothened agonist, and Purmorphamine) have been shown to successfully transdifferentiate human astrocytes into neurons [77]. 

In 2015, the direct conversion of fibroblast to cardiomyocytes was done using a combination of only chemical compounds (CRFVPT: CHIR99021, RepSox, Forskolin, VPA, Parnate, TTNPB) [78]. In 2016, the same transdifferentiation was shown to be successful by Cao et al. but this time using only a combination of 7 small molecules named the 7C combination (CHIR99021, A83-01, BIX01294, AS8351, SC1, Y27632, an OAC2) [79]. A compound, BRD7389, was shown by Xie et al. to be able to cause a pancreatic Alpha cell to adopt morphological and gene expression features of Beta-cells [80].

### 2.3. Epigenetic Modification

The process of direct cell reprogramming involving various epigenetic mechanisms are yet to be understood. The major epigenetic modification that regulate gene expression profiles includes histone modifications, chromatic remodeling, DNA methylation and ncRNAs [81]. The change in the chromatin structure (compact vs. loose) can subsequently changes the accessibility of the transcriptional machinery to the DNA template thereby affecting the regulation of transcription.

Histone modifications play a major role during cell fate and conversion by regulating the expression of certain key genes and can either act as a barriers or stimulators during the process of transdifferentiation [82]. This represents an opportunity since targeting these modifications and the enzymes can help improve the efficiency of the direct reprogramming.

In 2013, Barneda-Zahonero et al. demonstrated that the expression of histone deacetylase HDAC7 was induced in pre-B cells but reduced during the conversion of these cells into macrophages [83]. This was then shown to be due to the fact that HDAC7 depresses macrophage-associated genes and favors macrophage induction [83]. In 2016, the epigenetic regulator, Bmi1, a polycomb group protein, was shown to plays a major role in blocking the direct conversion of fibroblasts into cardiomyocytes [84]. 

Using small molecules targeting histone modification and signaling pathways were also shown to induce the process of transdifferentiation. In 2013, Bramswig and colleagues, demonstrated that controlling the histone methylation levels using an unspecific histone methyltransferase inhibitor Adox was capable of converting pancreatic α cell into β cell [85].

### 2.4. MicroRNA

MicroRNAs (miRNAs) are small non-coding RNA molecules composed of 18 to 25 nucleotides known to regulate the expression of several genes [86]. They are involved in altering the cell fate and have been involved in the process of transdifferentiation [48,87,88,89,90].

In 2011, Yoo and colleagues transfected fibroblasts with mir-9/9* and mir-124 and observed the expression of neuronal markers 4 weeks after [90]. However, it has been shown that it is easier to promote the conversion into defined subtypes of neurons using TFs than using miRNAs [91,92].

In 2012, Jayawardena and colleagues used a cocktail of miRNAs consisting of mir-1, 133, 208, and 409, to convert fibroblasts into myocardial cells [88]. This was performed with a transfection method. Later the same group has shown the use of lipid based method to transfect chemically modified miRNAs into these cells to be highly efficient [88,93].

### 2.5. Ribosomes

Ribosomes are complex biological macromolecule that contain a combination of several ribosomal proteins and RNAs [94,95]. Ribosomes play a major role in translation. They have also been shown to play key roles during cell development and differentiation [96].

The study of lactic acid bacteria (LAB) in the conversion of human dermal fibroblasts (HDFs) [97] have led to the identification of the role of ribosomes in the transdifferentiation process [98]. They have shown that ribosome incorporated cell clusters (RICs) are capable of transdifferentiation in vitro into ectodermal neurons, mesodermal cardiomyocytes, and endodermal hepatocytes.

### 2.6. Proteins and Cell Penetrating Peptides

The idea that cell reprogramming can be promoted using proteins is not new. In theory, cell extracts comprise the nuclear regulatory components which are needed to drive cell fate change.

In 2002 and 2003, two group have shown that the transdifferentiation of adult cells was possible by incubating them with extracts of either primary or transformed T-cells, or neuronal progenitor cells [99,100,101]. The problem with the latter was that they were macromolecules thus, their penetration via the plasma membrane was not efficient. Early work on human immunodeficiency virus showed that a protein trans-activator of transcription (HIV-TAT) could fuse with the plasma membrane to enter into the cells [102]. It was followed by the identification of other peptides know to penetrate the cell membrane [103,104]. These peptides were named cell-penetrating peptides (CPPs). They are also known as protein transduction domains (PTDs).

C-terminal fused undeca-arginine (11R) have also been routinely used for purpose of transdifferentiation. Erythroblastosis virus E26 oncogene homolog 2 and mesoderm posterior 1 fusion proteins have been shown to successfully reprogram HDFs into cardiac progenitors [105].

The conversion of human adipose-derived stem cells (ADSCs) into corneal endothelia (CE)-like cells was carried out with the help of recombinant TAT-transcription factors and small molecules [106,107]. The combination of CPP C-end rule (CendR) with Sox2 have been shown to differentiate pigmented epithelial (RPE) cells into functional neurons [106,107]. HDFs have also been converted into cardiac progenitor cells using QQ-reagent (a protein transduction reagent) [76].

## 3. In-Silico Direct Reprogramming Factor Prediction

Cell differentiation can be controlled via a small number of ectopic factors including TFs, micro RNAs, epigenetic re-modelers [108], growth factors and small molecules [78,79,109]). Many key factors involved in transdifferentiation were identified largely based on “trial and error” approach using experimental procedures. However, this is extremely time-consuming and inefficient in some cases. To address this issue, several groups have reported on the development of computational prediction algorithm which offer a better starting point for a large number of cell conversion study [24,25,27,28,110,111,112,113]. Here, we briefly review three methods whose in-silico TF predictions have been experimentally validated via human cell conversion: D’Alessio et al. [25], Mogrify [27], CellNet [24,111,112], and a recently developed algorithm: Ronquist et al. [113] which predicts not only transcription factors but also the amount and timing for the introduction of these factors.

Most methods consider a cell-type specific gene expression profile as a representation of cell identity when predicting which TFs are required to acquire the desirable cell. For example, D’Alessio et al. utilized massive microarray dataset for ~200 cell and tissue types primarily from the Human Body Index collection from GEO (Gene Expression Omnibus, GSE7307), which is one of the largest repositories of gene expression [114]. They hypothesized that if gene expression pattern of a certain TF matches the ideal expression pattern (where the TF is highly expressed only in a target cell and not expressed in any of the background cells), the TF is important to acquire the desired cell type. The background cell-types were selected based on Pearson correlation coefficients of expression profiles and the final score for TFs were calculated by using an entropy-based score using Jensen–Shannon divergence (JSD: The score measures the deviance of the ideal expression pattern from the observed) [25].

Although gene expression alone provides rich information on cell identity, genes do not work independently. Therefore, the transcriptional impact of TFs should also be estimated in the context of gene regulatory network (GRN). In Mogrify and CellNet algorithms, they used GRN information in addition to the gene expression profile.

Mogrify analyzed CAGE-seq (cap analysis of gene expression sequencing) data for ~300 cell and tissues from FANTOM5 consortium [115] and incorporated the existing network information from STRING database [116] and MARA (Motif Activity Response Analysis) [117]. They ranked two types of scores for each TF and aggregated the rankings into one consensus ranking. First type of score was the differential expression score which was calculated by combining the adjusted *p*-value and log fold change from the comparison between target and background cell-types. The background cell-types were selected based on the FANTOM5 cell-type ontology tree for each target cell. The other type of score was the network score which was computed as the weighted sum of differential expression scores of all interacting genes with a TF in the network. Each differential expression score in the network was weighted according to the number of co-regulated genes (to penalize TFs associated with too many genes) and the distance from the TF of interest (to consider that direct regulation is more effective than indirect). Mogrify outputs the highly ranked TFs after filtering out TFs already expressed in the starting cell type and TFs redundant in their influence in the network to get maximized regulatory coverage [27].

In the case of CellNet, it was originally designed to work with microarray data for ~20 cell-types [24,111] but recently it has expanded its applicability to use RNA-seq data and has been made publicly available for users to predict new cell conversion based on their own gene expression data [112]. To prioritize TFs for cell conversion, they devised a network influence score which was calculated by using the degree of dysregulation of the TF and its target genes in the cell type-specific GRN. The dysregulation of each gene was estimated based on the z-score determined by differential gene expression between the starting and target cell types, which was weighted by the expression level in the target cell type so as to allow more highly expressed genes to have a greater impact on the GRN status. The cell type-specific GRN was constructed in a two-step process. First, they created GRN using CLR (context likelihood of relatedness) algorithm, in which each edge between two genes was detected by correlation or MI (mutual information; it detects statistical dependency of the gene expression pattern between two genes and better captures nonlinearity of the relatedness than correlation) score. Second, they split up the large single GRN into multiple subnetworks using InfoMap community-detection algorithm [118] and combined them into one general GRN per cell type based on gene set analysis [119].

Recent advances in DNA sequencing technology allowed us more complex modelling using diverse genetic and epigenetic information. For example, Ronquist et al. incorporated time-series fibroblast RNA-seq and TAD (Topologically Associating Domain) information from 3-dimensional DNA interacting capturing (Hi-C seq) data [113]. They conducted dimension reduction based on the TADs to minimize complexity in high dimensional genetic data and modelled the dynamics of the TAD-level expression over time. Then, they estimated the final cell state after TF addition based on the state transition matrix (approximated from the time-series data) and TF influence information which was inferred from TF-DNA binding sites, TF activity and gene accessibility data (DNase-seq data of human fibroblast). Finally, they determined the optimal amount and timing of the addition of TFs by minimizing the distance between the target and final state for the conversion from fibroblast to multiple cell-types.

This sophisticated algorithm is applicable only when the relevant data for cell-type of interest is available such as the synchronized time series expression and DNase-seq data. The more complex data for a wide range of cell-types become available, the more advanced algorithm would be applied to the prediction for many cell-types.

In addition, most algorithms so far have been applied to gene expression from bulk cell populations or tissues instead of using homogeneous population of a single cell type. Thus, employing single-cell RNA-seq is needed to solve the problem of tissue heterogeneity. This achievement will not only increase the possibility of discovering new cell conversion, but also provide a deeper insight into how the direct cell reprogramming process is controlled.

## 4. Major Applications of Direct Cell Reprogramming

Direct reprogramming of mature cells from one state to another brings with it a tremendous potential for using this technology in various fields. One of the most important application of transdifferentiation is tissue engineering.

In 2011, Huang P et al., have successfully reprogrammed hepatocytes to regenerate the liver in mice [57]. In 2012, Margariti et al., directly reprogrammed endothelial cells into vascular lumen with a typical endothelial morphology [120]. In 2017, Hong et al., succeeded in the conversion of human smooth muscle cells into endothelial lineage going by an intermediate vascular progenitor phase [121]. Ni et al., succeeded in creating transdifferentiated hepatocytes capable of fully performing an enhanced excretion of bile acids. It created treatments for cholestatic diseases where the liver is unable to move bile to the small intestine on its own as well as for liver diseases that are not just related to liver damage [122].

Aside from tissue engineering, regenerative medicine is the next field to benefit the most of direct reprogramming technologies. The three main barriers of the natural cell that call for the use of transdifferentiation are that (i) the target cell type does not proliferate as fast and efficient as required, (ii) are rare in the body, (iii) or are hard to regenerate. Perhaps neuronal cells are the most attractive cell type to be targeted by transdifferentiation technology as they fit in all the above categories.

The use of lentivirus poses the main issue with translating reprogrammed neuronal cells to the clinic. All neurodegenerative diseases are characterized by defects in neuronal or glial cells, which lead to condition like strokes and Parkinson’s diseases. The latter is characterized by the death or defects in dopamine-producing neurons in the brain leading to a drop in the dopamine levels and thus an abnormal activity of the brain [123,124].

In 2017, Tang and colleagues transdifferentiated Stroli cells into dopaminergic neurons showing high functionality and a therapeutic potential. The transplanted cells in Parkinson’s mouse model showed reduction in the size of the lesion while promoting the recovery of the fine motor and sensory neurons [125].

## 5. Challenges and Prospective Solutions for Integrating Direct Cell Reprogramming in Clinics

Despite promising technological advancements in direct reprogramming, several major difficulties and challenges still exist in translating this technology to clinical applications. The most evident issue is the use of lentiviruses that has potential risk of mutagenesis [126]. These mutations, even though rare, can be causative of cancer [127]. While a compromise can be made by using non-integrating viruses, which do not integrate in the host genome, these may induce a lesser risk of mutagenesis but do not deliver the same efficiency of transdifferentiation. The use of CRISPR/Cas9 reduces the risk of mutagenesis.

Keeping in mind that the transdifferentiated cells may not be capable of mimicking the morphology of the target cell, their inability to perform their functions remain a major concern. Although in vitro experimentations allow for the study of the major characteristics of the reprogrammed cell, in vivo assays are compulsory to fully characterize the reprogrammed cells in their physiological setting. Tight regulation of in vivo assays along with extensive animal testing is key to the translation of direct cell reprogramming to clinical applications.

Finally, the efficiency of transdifferentiation remains a challenge. Low conversion rates mean that a longer period of time is needed before the desired yield of the reprogrammed target cells for any clinical application is reached. Thus, improving the efficiency and the yield are vital for making transdifferentiation favorable in clinical setting which are usually time-sensitive.

The above can be overcome via a myriad of methods including the optimization of biochemical [128], biophysical [129] and biomechanical [130] conditions the cells undergo during the process of transdifferentiation.
